# EIT monitors valid and robust regional ventilation distribution in pathologic ventilation states in porcine study using differential DualEnergy-CT (ΔDECT)

**DOI:** 10.1038/s41598-019-45251-7

**Published:** 2019-07-05

**Authors:** Sebastian D. Reinartz, Michael Imhoff, René Tolba, Felix Fischer, Eike G. Fischer, Eckhard Teschner, Sabine Koch, Yvo Gärber, Peter Isfort, Felix Gremse

**Affiliations:** 1 Department of Diagnostic and Interventional Radiology, University Hospital, RWTH Aachen University, 52074 Aachen, Germany; 2 Department for Medical Informatics, Biometry and Epidemiology, Ruhr University of Bochum, 44780 Bochum, Germany; 3 Institute of Laboratory Animal Science, University Hospital, RWTH Aachen University, 52074 Aachen, Germany; 4 Drägerwerk AG & Co. KGaA, Moislinger Allee 53-55, 23558 Lübeck, Germany; 5 Aix Scientifics CRO, Theaterstr. 7, 52062 Aachen, Germany; 6 Institute for Experimental Molecular Imaging, University Hospital, RWTH Aachen University, 52074 Aachen, Germany

**Keywords:** Three-dimensional imaging, Experimental models of disease

## Abstract

It is crucial to precisely monitor ventilation and correctly diagnose ventilation-related pathological states for averting lung collapse and lung failure in Intensive Care Unit (ICU) patients. Although Electrical Impedance Tomography (EIT) may deliver this information continuously and non-invasively at bedside, to date there are no studies that systematically compare EIT and Dual Energy CT (DECT) during inspiration and expiration (ΔDECT) regarding varying physiological and ICU-typical pathological conditions such as atelectasis. This study aims to prove the accuracy of EIT through quantitative identification and monitoring of pathological ventilation conditions on a four-quadrant basis using ΔDECT. In a cohort of 13 pigs, this study investigated systematic changes in tidal volume (TV) and positive end-expiratory pressure (PEEP) under physiological ventilation conditions. Pathological ventilation conditions were established experimentally by single-lung ventilation and pulmonary saline lavage. Spirometric data were compared to voxel-based entire lung ΔDECT, and EIT intensities were compared to ΔDECT of a 12-cm slab of the lung around the EIT belt, the so called ΔDECT_Belt_. To validate ΔDECT data with spirometry, a Pearson’s correlation coefficient of 0.92 was found for 234 ventilation conditions. Comparing EIT intensity with ΔDECT_(Belt)_, the correlation r = 0.84 was found. Normalized cross-correlation function (NCCF) between scaled global impedance (EIT) waveforms and global volume ventilator curves was r = 0.99 ± 0.003. The EIT technique correctly identified the ventilated lung in all cases of single-lung ventilation. In the four-quadrant based evaluation, which assesses the difference between end-expiratory lung volume (ΔEELV) and the corresponding parameter in EIT, i.e. the end-expiratory lung impedance (ΔEELI), the Pearson’s correlation coefficient of 0.94 was found. The respective Pearson’s correlation coefficients implies good to excellent concurrence between global and regional EIT ventilation data validated by ventilator spirometry and DECT imaging. By providing real-time images of the lung, EIT is a promising, EIT is a promising, clinically robust tool for bedside assessment of regional ventilation distribution and changes of end-expiratory lung volume.

## Introduction

Electrical Impedance Tomography (EIT) is a non-invasive bedside monitoring technique, that uses an electrode belt stretched around the thorax to measure the intrathoracic resistivity and its changes during breathing. The resistivity increases the more the alveoli are filled with air because the electrical current must flow through increasingly thinned and elongated interalveolar septa. EIT allows monitoring of the respiratory cycle with regard to harmful alterations such as atelectasis, pneumonia or pneumothorax as well as emphysema or volutrauma caused by overdistension due to inappropriate mechanical ventilation settings^1^. The EIT output consists of the temporal changes of the cumulative resistivity changes as a so-called EIT plethysmogram and a spatially coded overview of the resistivity changes as a so-called ventilation map in the sense of a tomogram, which therefore monitors global ventilation and displays regional ventilation distribution in a cross-section of the lung in real time^2^. The latter aspect is particularly important for patients with mechanical ventilation on an intensive care unit (ICU): gravity-dependent lung collapse or atelectasis induces pathologic ventilation distribution of the lung which is generally not reflected in changes in global ventilation parameters or airway pressure spirometry data. Additionally, respirator-induced repeated reopening of collapsed small airways^3–5^ airways leads to shear stress for the alveolar walls. Consequently, alveolar collapse and hypoventilation may exist alongside alveolar overdistension. As a result, ventilator-induced lung injury (VILI) can clinically develop virtually unnoticed. The only hint could be an increased tidal recruitment that is known to be an independent risk factor for death on the ICU^6^. On the other hand, ineffective oxygenation due to pendelluft may take place. As a prophylaxis, an optimal PEEP (positive end expiratory pressure) level in conjunction with limited inspiratory pressures and tidal volumes (TV) counteract the development of VILI, which may be identified individually by a so-called PEEP trial with recruitment maneuvers. Finding and maintaining these crucial settings during ICU therapy is challenging. Monitoring regional ventilation distribution can significantly facilitate the detection of regional ventilation imbalance^7–9^ that may lead to atelectasis and VILI as well as the identification of the optimal PEEP level^8,10^. On the bedside, chest ultrasound (US) is a very useful point-of-care technique to quickly evaluate fluid collections like pleural effusion as well as consolidating alterations of the lung, like atelectasis and pneumonia. A general limitation is that ventilated lung structures cannot be visualized due to total reflection of sound waves at the tissue-air-border. In contrast to EIT, the US device has to be attached manually for each measurement and does not provide a continuous overview of the whole lunge. Even though chest x-ray, CT and magnetic resonance imaging (MRI)^11^ are powerful and established diagnostic methods for assessing lung-protective ventilation, they do not provide the possibility for long-term continuous recording at the bedside of patients on the Intensiv Care Unit (ICU).

Computed tomography (CT) is the gold standard for assessing global and regional aeration^12^ in healthy and diseased lungs^13^, for example, for detecting pneumonia, pleural effusion, pneumothorax or atelectasis. In clinical routine, however, CT offers only a static momentary ‘snapshot’ view of the pulmonary status. Moreover, CT also exposes the patient to ionizing radiation and to risks associated to intra-hospital transport.

In contrast, global spirometry data, which reflect the condition of the lung as a whole, cannot detect focal pathologies such as atelectasis or pleural effusion with sufficient certainty. Due to these drawbacks of both techniques, CT and spirometry, the continuous assessment of regional ventilation distribution — namely, of how different lung regions respond to therapeutic interventions over time — is mostly unclear in clinical routine.

Until now, only one study^10^ has performed differential CT (ΔCT), i.e. CT in end-inspiratory and end-expiratory breath holds for comparison purposes. This calculation of ΔCT provided dynamic information about regional ventilation distribution as well as information on global and regional air volume changes, using a slice thickness of 2–8 mm. To address this lack of validation data our study investigated typical pulmonary pathologies in a porcine model resembling clinical scenarios observed in ICU patients. Pathologies were monitored using EIT and compared with simultaneously acquired global ventilation data (ventilator spirometry) and sequentially acquired ΔCT using DualEnergy Technique (ΔDECT) for artifact elimination, which emerged by the EIT-belt. Healthy lungs, gross changes in ventilation distribution (single lung ventilation) and more discrete changes in ventilation distribution (lavage-induced atelectasis) were assessed at different ventilator settings before and after recruitment maneuvers. Global and regional EIT data were compared to ventilator spirometry and global and regional ΔDECT data.

## Results

### Device and image datasets

A total of 468 VMC DECT datasets, representing approximately 220,000 CT slices, and 234 EIT ventilator datasets were evaluated. None of the data was missing or corrupt. Dose length product (DLP) averaged 20,947.3 ± 7,531.93 mGycm for each animal. Using a moderate conversion factor of 0.015, it represents 314.2 ± 113.0 mSv in total.

### Analysis of global ventilation

**Figure 1:**
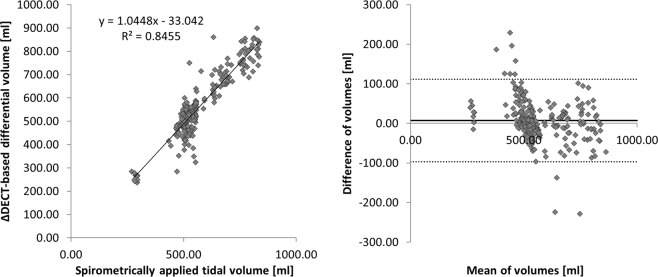
Pearson’s correlation coefficient regarding tidal volumes between spirometrically and ΔDECT-based analysis yielded r = 0.92.

On average 1,370 ± 262 ventilator and EIT signal datasets were compared for each experiment. Overall, the applied tidal volume by the ventilator was 577.58 ± 118.75 ml. In 234 ΔDECT of the whole lung, the mean tidal volume was calculated as 570.42 ± 134.93 ml. The mean absolute difference in tidal volume between spirometry and ΔDECT is computed to 36.74 ± 39.27 ml, meaning approximately 6.4% of the spirometrically applied tidal volume. The Pearson’s correlation coefficient was 0.92 between ΔDECT and spirometrically detected tidal volume (Fig. 1).

Comparing EIT intensity with belt-adjusted ΔDECT_,_ the so-called ΔDECT_(Belt),_ for n = 234 experiments, the mean EIT intensity was 2,592.96 ± 834.23, representing 208.73 ± 78.94 ml in ΔDECT_(Belt)._ This leads to a correlation coefficient of r = 0.84.

Vice versa, the normalized cross-correlation function (NCCF) between scaled global impedance (EIT) waveforms and global volume ventilator curves of the 13 animals was 0.99 ± 0.003.

### Analysis of regional ventilation

In experimental step D21 representing one-sided lung ventilation, the right lung was solely ventilated in 77% (n = 10) of the cases, which was validated by ΔDECT imaging. In the other three cases, left-lung ventilation was performed.

**Figure 2:**
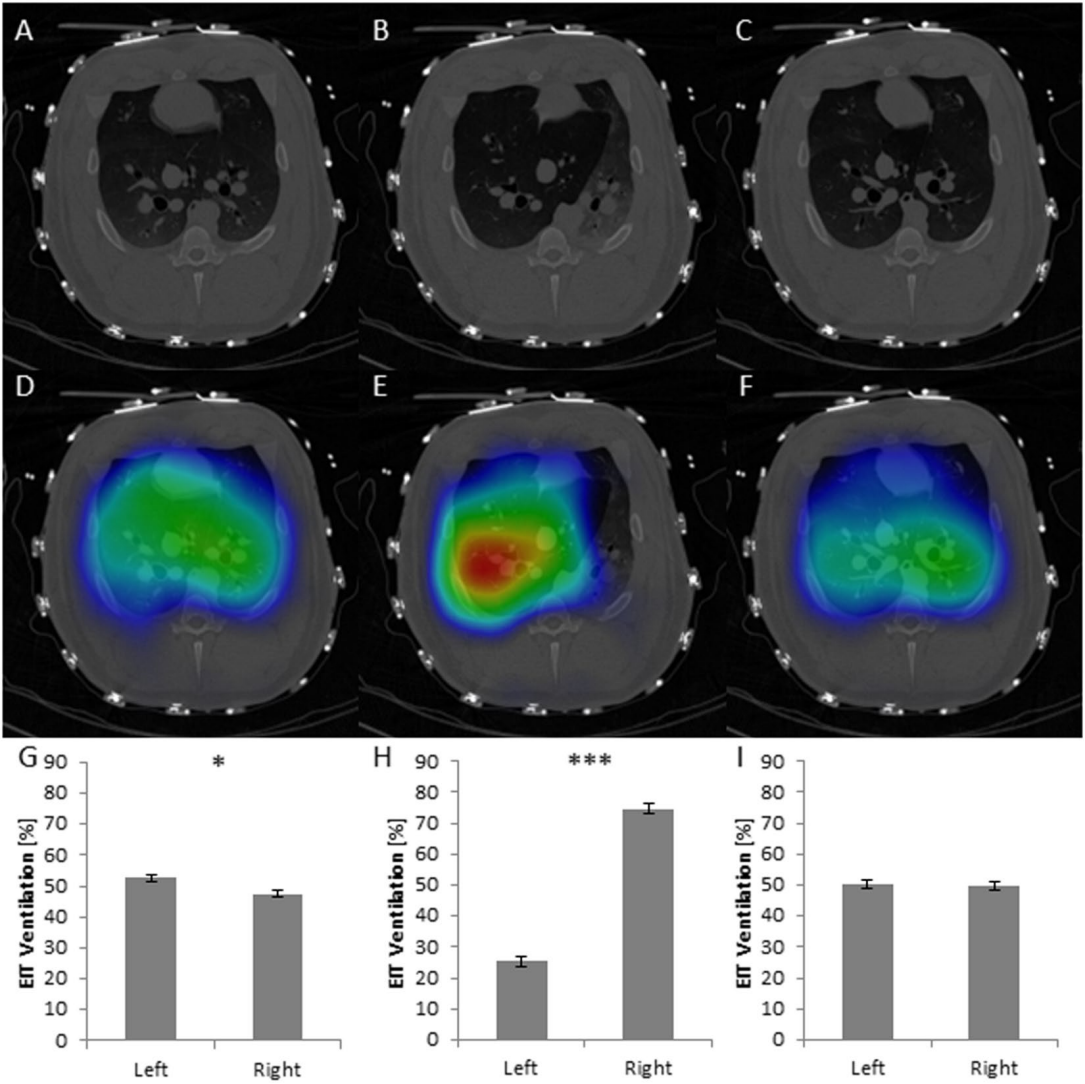
Example for right-sided ventilation, detected in EIT. Lines: DECT, DECT with EIT overlay, column diagram with EIT-based percentual ventilation of left and right quadrants. First row (**A**,**D**,**G**) shows native DECT with orthotopically positioned tracheal tube, homogeneous EIT signal and almost side-leveled percentual ventilation in EIT signal analysis. After propagation of tracheal tube in right-sided main bronchus, DECT (**B**) demonstrates left-sided atelectasis and right-sided hyperinflation which exceeds over the center line. Correspondingly, intense EIT signal (**E**,**H**) is seen in hyperinflated areas whereas either no or massively reduced signal intensity is detected in atelectasis areas. After restitution to tracheal position, (**C**), normal EIT signal intensities (**F**) and ratios (**I**) are evident again. In every wrong tube position, EIT signal analysis demonstrated significant changes in EIT signal intensity ratios.

In the case of right-sided ventilation, the mean percentage signal values were 74.78% ± 5.9 on the ventilated side. In contrast, if ventilation was established on the left side, the mean values yielded 92.49% ± 7.4 respectively. After restoring two-sided ventilation in experimental step D22, the mean values were 51.99% ± 1.4 for the left and 48.01% ± 1.4 for the right lung. In any case, this situation differed statistically significantly from two-sided ventilation for every animal. An example is shown in Fig. 2.

**Figure 3:**
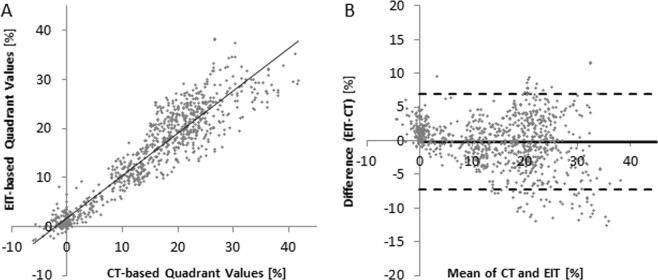
For comparison of EIT with ΔDECT_(Belt)_, quadrant-based ΔEELI and ΔEELV values were correlated, yielding a Pearson correlation coefficient of r = 0.94. A Bland-Altman plot shows a systematic overestimation of small values and underestimation of large values by EIT compared to ΔDECT_(Belt)_.

For comparison of EIT with ΔDECT_(Belt),_ especially in slow recruitment maneuver (D31-D37), quadrant-based ΔEELI and ΔEELV mean values were 13.67 ± 10.79 vs. 13.46 ± 9.94. The analysis yielded a Pearson correlation coefficient of r = 0.94. A Bland-Altman plot shows a systematic overestimation of small values and underestimation of large values by EIT compared to ΔDECT_(Belt)_ (Fig. 3).

## Discussion

The aim of this study was to systematically compare EIT with spirometry and CT under ICU-specific physiologic and pathologic ventilation conditions. While ventilator data was compared with CT of the entire lung, EIT was compared to CT restricted to the belt region_._ In order to validly extract EIT- and spirometry-compatible parameters such as tidal volume and regional air distribution from the CT data set, a CT protocol meeting specific requirements was selected. That play a decisive role in image acquisition, image processing and image interpretation. Large metal electrodes in the belt cause strong streaking artifacts, which significantly hinder an artifact-free evaluation of the lung region, even by using iterative reconstruction techniques^14,15^, which are used for artifact reduction. Due to the circular arrangement of the electrode belt, physical reduction of the absorption and scattering effects is not possible^16^. For this reason, artifact reduction was performed using Dual Energy technology^17,18^ which allowed generation of artifact-free virtual monochromatic image data^18–20^ by taking into account different fractions of Photon and Compton effect. Prior to this, the question must be answered whether the lung should be examined statically, i.e. at a certain point in time, e.g. end expiratory, or dynamically, i.e. 4D data acquisition. The latter involves repetitive irradiation of the examination section, which is used clinically for perfusion imaging of the brain or heart^21–23^. In order to enable a larger z-axis coverage even with larger detectors a permanent table movement between two adjacent detector positions^24^ takes place in the so-called shuttle mode. This, together with extremely wide detectors^25,26^ with more than 512 rows, which had not yet been developed at the time of the study, achieves a z-axis coverage of 14–30 cm, which does not meet the minimum requirement for full lung coverage (35–38 cm). Dynamic CT over 45 seconds has been used by Wrigge *et al*.^7^ during a slow inflation maneuver. Using given gantry rotation time of 0.5 seconds and a scanning duration of 4.5 seconds for each step with a 16 slice CT, z-axis coverage is approximately 14 centimeters, which does not represent the entire lung. Analogously, Yoshida^27^ used dynamic CT in one animal during continued artificial ventilation using “thick slices”. That means that the concept of dynamic whole-lung coverage by CT has not been realized yet. In this context, we decided to perform static CT acquisition using ventilation-enforced breath hold. By calculation of difference images, a dynamic information is provided in the sense of the study objectives such as tidal volume.

To completely capture the animal’s lung volume from apical to basal in the Field-of View, it had to be calibrated to the average diameter at the height of the diaphragm, which amounts to between 30–34 cm. It has to be considered that the pixel size is derived from the size of the FOV and the standard reconstruction matrix of 512 × 512 pixels. In order to achieve a three-dimensional image of the lung with cube-shaped, i.e. undistorted (isotropic), voxels the FOV was set to 380 × 380 mm. This results in a calculated in-plane resolution by 0.75mm^2^, which was completed to isotropic voxels in the 3rd dimension with a layer thickness of 0.75 mm (isotropic spatial resolution). Assuming^28^ that the volume of an acinus with approx. 2000 alveoli in end-expiratory phase is approx. 20 mm^2^, in this study a voxel volume of 0.42 mm^3^ has been generated, which contains only 42 alveoli in the so called CT pulmonary unit^28^. By using this differential DECT protocol with a detector collimation of 2 × 64 × 0.6 mm and a relatively slow table feed of 13.7 cm/s, an examination time of 2.5 to 2.9 seconds could be achieved by applying spiral CT technique. Until now, only Meier *et al*.^8^ used differential CT as reference method for preclinical EIT devices (Dräger Evaluation Kit 2) in a swine model with six animals, but with a non-isotropic resolution of 2 mm slice thickness. That means that regional ventilation distribution could be assessed in plane but with a significantly reduced resolution on a 3D basis. Furthermore, not the entire lung was scanned. Both aspects could be the reason for a wide range of correlation coefficients between ΔCT and EIT for tidal volumes (Range r: 0.55–0.88).

By means of this method, the tidal volume applied by the ventilator, was made visible three-dimensionally through differential ΔDECT with submillimeter isotropy, conducted in end-inspiratory and end-expiratory breath-hold. Vice versa, the EIT intensities were correlated with identical ΔDECT datasets, which were virtually restricted to a slab of 12 cm (ΔDECT_(Belt)_). This corresponds to the lung coverage expected by the EIT device.

For quantitative evaluation, the linear relationship between density in Hounsfield units of the voxel, i.e. the 42 air-filled alveoli, and tissue/liquids was exploited^29^. In particular, the distribution of the air and not of the tissue was evaluated in a manner similar to emphysema quantification^30^.

Using this high-spatial and temporal resolution concept, it was possible to excellently correlate the spirometry with ΔDECT datasets (r = 0.92). Furthermore, ΔDECT_(Belt),_ datasets also showed a good correlation of the EIT monitored lung area with r = 0.84. For validation purposes, spirometry and EIT intensities correlated excellently (r = 0.99 ± 0.003) in the direct comparison. That means, ΔDECT can be used as bridging modality between EIT and spirometry.

With regards to this and regional ventilation distribution, the correlation between EIT intensity and ΔDECT_(Belt)_ is lower than the analogous analysis utilizing ΔEELI and ΔEELV. This aspect is in line with the findings from Meier *et al*.^8^, who also computed a huge range of r (0.55–0.88) for tidal volume and r = 0.98 for EELV by using a differential CT between end-expiratory and end-inspiratory hold. A reason for this could be the focused lung volume observed by EIT, which is approximately six centimeters above and below the belt. Due to diaphragmatic breathing, the lung expands in caudal direction, and a relevant amount of the tidal volume disappears from the belt plane. This phenomenon was evident in ΔCT data of Meier *et al*., who referenced EIT data by a single CT- slice of 2 mm thickness in inspiratory and expiratory ventilation position. Under different PEEP settings, the diaphragm alters its position; thus, visibility of tidal volume (TV) in one representative CT slice varies. This being said, one can conclude that TV affects ΔDECT_(Belt)_ data, which correlates worse with EIT intensities. Consequently, tidal volume-cleared end-expiratory data (ΔEELV) correlates well.

Furthermore, in contrast to the aforementioned studies, the EIT device used in this study (Pulmovista® 500, Dräger, Lübeck, Germany) is commercially available and licensed to be used in clinical settings.

### Limitations

Our study concept cannot investigate every possible ventilation condition potentially arising in ventilated critically ill patients- therefore typical pathologies were investigated exemplarily in different levels of markedness. Pathologic settings like (tension-) pneumothorax, pneumonia or left heart failure with pulmonary edema are not represented in this study design.

Furthermore, the applied tidal volumes and PEEP settings are only representative for a continuous spectrum of settings and possible permutations, which can be found in real life. However, we are aware of the limitations of the preclinical pig model within this setting. But, to the best of our knowledge there is no other large animal model with a better correlation to the human situation.

Therefore, in this systematic trial using a clinically licensed EIT device (PulmoVista® 500, Dräger, Lübeck, Germany) in a large animal model, a causal relationship between spirometry of the ventilator and EIT data has been validated by ΔDECT for regional ventilation distribution and changes of lung volume under physiologic and pathologic ventilation settings (atelectasis and single-lung ventilation). Providing real-time identical images of the lung, EIT is a promising, clinically robust tool for bedside assessment of regional ventilation distribution and changes of end-expiratory lung volume.

## Methods

The datasets generated during and/or analyzed during the current study are available from the corresponding author on reasonable request and under acceptable conditions for the sponsor.

### Study design

This study is a non-blinded, prospective animal trial using a porcine model. EIT was correlated with ΔDECT, which was virtually restricted to a slab volume of 12-cm thickness aligned to the EIT belt for simulating EIT data in ΔDECT (ΔDECT_(Belt)_). On the other hand, the unrestricted ΔDECT-based tidal volume was compared to the spirometry data for comparing full regional ventilation information with the ventilator.

### Animal experiments

All pigs were non-pregnant female *sus scrofa domestica*, (breed: “Deutsche Landrasse”), purchased from Heinrichs Tierzucht GmbH (52525 Heinsberg, Germany).

Thirteen (13) healthy female pigs aged 140 (range 112–152) days were examined. Their weight was 66 kg (range 61–70 kg) at the day of experiment after one night of fasting. The circumference of the animals’ chests was 88 cm (range 84–91).

Upon delivery of the animals, veterinarian examination of general condition, weight, lung auscultation, and body orifices was performed and documented. As acclimatization period, the animals were housed for at least one week and received a standard diet (Ssniff pig diet, Ssniff Spezialdiäten GmbH, Soest-5% body weight per day) and water *ad libitum*. The experimental protocol was approved by the local government animal care and use authority, as explained in section “Contract Research Organization (CRO) and ethic competent authority” of methods. The animals were fasted for about 12 hours prior to the experiment with free access to water. Atropin 2 mg/kg (Dr. Franz Köhler Chemie GmbH, 64625 Bernsheim, Germany) and Azaperone 5 mg/kg (Stresnil (R); Lilly Deutschland GmbH, Elanco Animal health, 61352 Bad Homburg) were administered intramuscularly as premedication. Propofol 1 mg/kg (Propofol Claris 2%; PHARMORE GmbH, 49479 Ibbenbüren, Germany) was given as an intravenous bolus followed by tracheal intubation. Anesthesia was maintained by infusion of 35 µg/kg/h Fentanyl (Rotexmedica Arzneimittelwerk GmbH, 22946 Trittau, Germany) and propofol (8 mg/KG x h). Muscle relaxation was induced by Pancuronium (0.2 mg/ kg Bodyweight, and repeated every 60 min with 0.1 mg/kg). Arterial blood pressure was maintained by fluid infusion of Ringer lactate (Baxter, 3542 CE Utrecht, Netherlands) and Glucose 5% (Baxter, 3542 CE Utrecht, Netherlands). Monitoring throughout the experiment included ECG, arterial, central venous pressure, oxygen via pulse oximetry (Infinity Delta physiologic monitor, Dräger, Lübeck, Germany). Anesthesia was controlled by a veterinarian experienced in Laboratory Animal Science. Blood samples were collected from the jugular vein before induction of anesthesia using serum tubes (Fa. Sarstedt, 51588 Nümbrecht, Germany) for the measurements of hematological parameters. All preparations were conducted under aseptic conditions.

The swine was positioned in a supine position on the CT table on a deflated vacuum mattress and the animal was instrumented for the monitoring of CO_2_, SpO_2_, invasive arterial blood pressure, central venous pressure, body temperature, intermittent blood gas analysis, ECG, and urine production. After positioning, the correct position of the EIT electrode belt, which is at the height of the axilla with the two indicator electrodes positioned left and right of the sternum, was verified by an initial thoracic CT scan. Then, body position was fixed by activating the vacuum mattress. The body temperature of the animals was held constant by external warming through a heat blanket.

The entire study was conducted to and performed in compliance with the European Directive 2010/63/EU, the German law on Animal Protection (Tierschutzgesetz), the Guide for the Care and Use of Laboratory Animals (USA), and according to the Japanese Ordinance 37 (2005) of the Ministry of Health on GLP for Nonclinical Safety Studies of Medical Devices.

### Experimental setup

**Figure 4:**
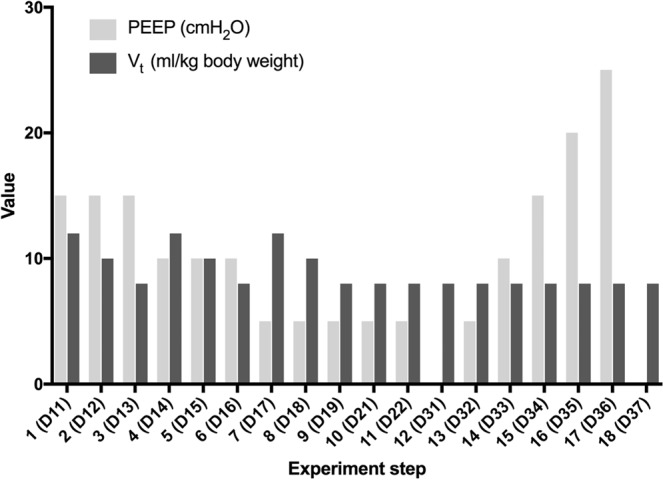
Overview of experimental steps: Decremental PEEP in Section 1 (D11-D19) with each varying tidal volume, one lung ventilation in Section 2 (D21-D22) and slow recruitment maneuver in Section 3 (D31-D37). PEEP in cmH_2_O, Vt in ml/kg body weight.

The experiment for each animal consisted of 18 standard or pathological ventilation situations (Fig. 4); for each setting, ΔDECT scans were performed to be compared with the corresponding EIT images. The 18 settings, which were grouped into three parts, are as follows:

In part 1 of the experiment three different PEEP settings, decreasing from 15 cmH_2_O over 10 to 5 cmH_2_O were evaluated. On each PEEP level, the tidal volume (TV) was decreased from 12 over 10 to 8 ml/kg body weight (D11-D19). These nine settings represent different physiological states of ventilation (Fig. 4).

In Part 2, the final settings of part I for TV (5 cmH_2_O; 8 ml/kg body weight) and PEEP were continued, but a one-sided lung ventilation was established (D21) by advancing the endotracheal tube into one main bronchus. Afterwards, the two-lung ventilation was re-established (D22) by repositioning of the tube. These cases simulate rough regional ventilation disorders represented by one-sided lung ventilation.

In Part 3, an artificial atelectasis and lung injury was induced by repeated intrapulmonary saline lavage (approximately 2.0–6.0 liters total lavage volume) with the addition of up to 1.5 liters of 6% hydroxyethyl starch solution (HES) for depletion of lung surfactant. PEEP was set to 0 during lavage. Then six examinations with PEEP values ranging from 0 to 25 cmH_2_O in 5 cmH_2_O increments were conducted to represent a slow recruitment maneuver (D31, then D36-D32). In the final examination, the PEEP was set to 0 cmH_2_O again for comparison with step D31 (Fig. 4). One pig suffered from a tension pneumothorax during the intrapulmonary saline lavage as an adverse event. This was treated by CT-guided chest drainage. Afterwards, the experiment could be continued and finalized in accordance with the investigation plan.

After completion of the experiment, animals were sacrificed by an intravenous overdose of Pentobarbital.

### Measurements and evaluation procedures

#### EIT device

The EIT device (PulmoVista® 500, Dräger, Lübeck, Germany) is described elsewhere in detail^31–33^. Briefly, during the entire experiment, the EIT data was recorded with 50 frames per second. The device was sealed during onset of the study to render software changes impossible. It reconstructed Tidal images as well as ΔEELI images, which represent the regional distribution of the end-expiratory ventilation changes. In clinical use cases, as well as in this study, ΔEELI images are computed and shown for a sequence of decremented PEEP values.

#### CT

All CT examinations were performed with a second generation Dual Source CT (DSCT, Siemens Definition Flash, Forchheim, Germany) using a dedicated Dual Energy protocol for metal artifact reduction of the EIT belt electrodes. During data acquisition, the tube voltage was set to 100 kV and 140 kV with a collimation of 2 × 2 × 64 × 0.6 mm. The tube current was set to maximum, approximately 300 mAs and 230 mAs, respectively, to provide scan duration of less than four seconds. The pigs were examined from above the thoracic apex to below the bottom of the dorsal costodiaphragmatic recess of the lung, to ensure covering the entire lung parenchyma. For each experiment step, an end-inspiratory as well as an end-expiratory lung scan was performed in breath-hold position.

The acquired raw-data was reconstructed using a hard convolution kernel (B50f) combined with a lung window, a 0.75 mm slice thickness, an increment of 0.6 mm and a field of view of 380 × 380 mm (matrix: 512 × 512 pixel) for each tube voltage. During post-processing, both datasets with different spectra (100 and 140 kV tube voltages) were merged on a dedicated workstation (DualEnergy, MMWP-Multimodality Workplace, Leonardo, Siemens, Forchheim) and virtual monochromatic images (VMC) with visually assessed minimal artifact burden of the lung were calculated. Only VMC data was evaluated with regard to study aims.

#### Ventilator

Animals were mechanically ventilated with the Evita ®XL ventilator (version 7.01, Dräger, Lübeck, Germany) using volume-controlled ventilation. Ventilation parameters were set per protocol and respiratory parameters (airway pressures, tidal volume, flows) were continuously monitored by the ventilator. To avoid unnecessary radiation exposure of the study personnel, end-expiratory and end-inspiratory hold maneuvers were performed by remote control. Vital signs (invasive blood pressure, ECG data, SpO_2_, etCO_2_ and body temperature) were continuously monitored (Infinity Delta physiologic monitor, Dräger, Lübeck, Germany).

All set and measured parameters from EIT, ventilator, and physiologic monitor were electronically recorded on dedicated study notebooks using commercially available software packages (Dräger, Lübeck, Germany).

### Automated data evaluation

For comparison with spirometric data, the TV of the entire lung was determined based on the ΔDECT datasets. To compare ΔDECT data with EIT data, end-inspiratory and end-expiratory gas volumes (EELV) and, thus, tidal volumes using a region of interest (ROI) were restricted to the belt region called ΔDECT_Belt_. This ROI was a slab centered to the belt plane and empirically set to twelve centimeters thickness, which was decided before starting the study.

**Figure 5:**
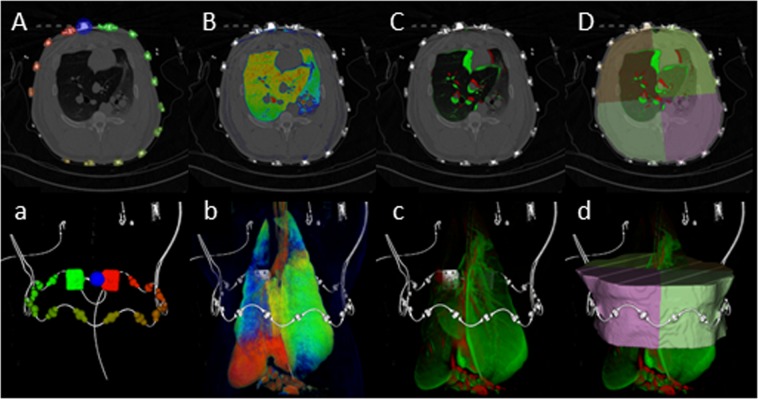
Automatic process of coregistration shown by axial slices view (capital letters **A–D**) and volume renderings (a–d): Step 1 consists of identification of the EIT belt electrodes in the CT datasets (**A**,a), which can be identified in the EIT data as well. Then pulmonary density maps (**B**,b) of inspiration and expiration were calculated. Consecutively, difference maps (**C**,c) were computed. Finally, analysis was virtually restricted to the volume next to the belt (**D**,d), which was called ΔDECT_(Belt)_.

For calculating regional tidal values, the twelve centimeter slab was divided into four quadrants (ventral left/ right and dorsal left/ right) based on the locations of the electrodes of the EIT belt. This segmentation was performed automatically centered to the belt for both modalities by dedicated software^34^. An orthogonal affine transformation was computed to match the electrode positions to the positions of an ideal virtual reference geometry, where the electrodes were placed in equidistant positions. The segmentation is defined as a label mask, which assigns each voxel to one of the four quadrants or “unclassified” (Fig. 5).

For a single voxel, the containing air volume can be estimated by its CT intensity because of the linear relationship between air content and density in Hounsfield units (HU). For example, voxels representing −1000 HU contain 100% air, voxels with −500 HU contain 50% air, and voxels with intensity equal or greater than 0 HU contain 0% air. The total air volume of a segmented region was computed by summing up the voxels of the region. The tidal volumes were computed by subtracting the end-expiratory values from the end-inspiratory values.

Analysis of the EIT signal was performed similar to the CT data sets by computing an orthogonal affine transformation between the EIT space and the virtual reference geometry. Then the reference segmentation was transformed into the EIT space and used to evaluate regional values of the four quadrants. The same quadrant definition for CT as for EIT was used to avoid systematic error.

Regional ΔEELI values were computed as well by using the segmentation of the four quadrants. Based on corresponding CT datasets, regional ΔEELV (differential end-expiratory lung volume) values were computed to assess the accuracy of the regional ΔEELI (differential end-expiratory lung impedance) values.

### Statistics

#### Case number calculation

The required case number was calculated for a required power of 0.9 for the correlation between global impedance changes and global tidal volumes.

#### General statistics

Global lung ventilation was assessed by cross-correlation function (CCF) between global impedance (EIT) waveforms and global volume (ventilator) curves. A phase shift between the two curves was considered, and both curves were normalized by subtracting the mean value and applying a scale factor so that the standard deviation is one. The arithmetic mean and the standard deviation of the R-values were determined. Furthermore, the upper and the lower 95% confidence levels were determined, and a one-sample-t-test was applied to test whether the mean of the R-values is significantly different from 0.9.

Regional lung distribution was tested by comparing left and right quadrants percentages of one-sided lung ventilation using a paired t-test. Dorsal ventilation quadrants of different states of atelectasis and full-lung ventilation were compared using one-way ANOVA and the Tukey post-test. Finally, the correlation coefficient r between these ΔDECT-based percentages and their corresponding EIT counterparts was calculated. For both ΔDECT and EIT scans, the percentages of the four quadrants were computed, i.e., the four quadrants sum up to 100%.

For corresponding regional ∆EELI and ∆EELV percentages (four values), the average absolute difference was computed, which represents a goodness score, i.e., the lower the better.

For ΔDECT, regional ∆EELV percentages were computed by subtracting regional air volumes of two end-expiratory CT scans with different PEEP values and subsequent normalization. Normalization was performed by dividing the values by the sum of the four regional values of the highest PEEP setting.

The significance threshold was assumed to be 0.05 for every evaluation. The SAS system software 9.4 was used for statistical calculations.

### Contract Research Organization (CRO) and ethic competent authority

A CRO (AIX-Scientific) was commissioned as a process quality assurance to ensure implementation of the preclinical investigation plan according to the Standard Operating Procedures and regulatory framework in a nonclinical safety study for medical devices as independent monitoring party. The entire study including image analysis was planned and the plan was documented in written form before starting the experiments to avoid any bias and to ensure the Quality according to the DIN ISO 13485. The study has been reviewed by the Competent Authority (LANUV: “Landesamt für Natur, Umwelt und Verbraucherschutz Nordrhein-Westfalen”, Registration Number: 84-02.04.2015.A170) and its Governmental Animal Care and Use Committee.

## Data Availability

Materials are to be distributed by a for-profit company (Draegerwerk AG & Co. KGaA, Lübeck, Germany). The data that support the findings of this study are available from Draegerwerk, but restrictions apply to the availability of these data, which were used under license for the current study, and so are not publicly available. However, the data is available either from the authors or from Draegerwerk itself (ClinicalAffairs@draeger.com) upon reasonable request and with permission of Draegerwerk.
